# Enhancement of ethanol production in very high gravity fermentation by reducing fermentation-induced oxidative stress in *Saccharomyces cerevisiae*

**DOI:** 10.1038/s41598-018-31558-4

**Published:** 2018-08-30

**Authors:** Thanawat Burphan, Supinda Tatip, Tossapol Limcharoensuk, Kitsada Kangboonruang, Chuenchit Boonchird, Choowong Auesukaree

**Affiliations:** 1 Department of Biology, Faculty of Science, Mahidol University, Bangkok, 10400 Thailand; 2 Center of Excellence on Environmental Health and Toxicology, CHE, Ministry of Education, Bangkok, 10400 Thailand; 3 Department of Biotechnology, Faculty of Science, Mahidol University, Bangkok, 10400 Thailand

## Abstract

During fermentation, yeast cells encounter a number of stresses, including hyperosmolarity, high ethanol concentration, and high temperature. Previous deletome analysis in the yeast *Saccharomyces cerevisiae* has revealed that *SOD1* gene encoding cytosolic Cu/Zn-superoxide dismutase (SOD), a major antioxidant enzyme, was required for tolerances to not only oxidative stress but also other stresses present during fermentation such as osmotic, ethanol, and heat stresses. It is therefore possible that these fermentation-associated stresses may also induce endogenous oxidative stress. In this study, we show that osmotic, ethanol, and heat stresses promoted generation of intracellular reactive oxygen species (ROS) such as superoxide anion in the cytosol through a mitochondria-independent mechanism. Consistent with this finding, cytosolic Cu/Zn-SOD, but not mitochondrial Mn-SOD, was required for protection against oxidative stress induced by these fermentation-associated stresses. Furthermore, supplementation of ROS scavengers such as N-acetyl-L-cysteine (NAC) alleviated oxidative stress induced during very high gravity (VHG) fermentation and enhanced fermentation performance at both normal and high temperatures. In addition, NAC also plays an important role in maintaining the Cu/Zn-SOD activity during VHG fermentation. These findings suggest the potential role of ROS scavengers for application in industrial-scale VHG ethanol fermentation.

## Introduction

Due to rapid industrialization and urbanization in many countries, global energy demand has dramatically increased in recent years. The rising consumption of fossil fuels leads to an increase in several environmental problems such as air pollution and global warming. The requirement of eco-friendly renewable fuels, especially bioethanol, is therefore increasing. The budding yeast *Saccharomyces cerevisiae* is commonly used in industrial ethanol production due to its abilities to efficiently produce ethanol from a broad range of biological substrates and tolerate numerous stresses present during fermentation^1^. During fermentation, yeast cells encounter a number of stresses, including hyperosmolarity, high ethanol concentration, and high temperature^2–4^. Upon pitching yeast cells into wort containing high concentrations of sugar substrates, yeast cells are suddenly exposed to osmotic stress. In addition, high ethanol concentration during fermentation is another important stress that affects not only yeast viability but also ethanol production^5^. Impact of osmotic and ethanol stresses are exacerbated when performing very high gravity (VHG) fermentation using media containing sugar concentration higher than 250 g L^−1^ sugar in order to achieve high ethanol yield (>15% (v/v))^6^. Although, in an industrial-scale ethanol production, the fermentation temperature is maintained by using cooling systems, heat generated by yeast metabolism and/or high environmental temperature could occasionally raise the fermentation temperature beyond an optimal range^7^. Heat stress is therefore another important stressor encountered by yeast cells. The effect of heat stress becomes more serious when performing fermentation at high temperature, a new strategy developed for gaining higher ethanol yield, decreasing the rate of bacterial contamination, and saving the cooling costs^8^.

Our previous deletome analysis has revealed that *SOD1* gene encoding cytosolic Cu/Zn-superoxide dismutase (SOD), one of important antioxidant enzymes, was required for tolerances to not only oxidative stress but also other stresses present during fermentation such as ethanol, osmotic, and heat stresses^9^. Among these fermentation-associated stresses, ethanol stress has been demonstrated to induce an increased generation of reactive oxygen species (ROS)^10^. Based on these findings, it seems that several fermentation-associated stresses may also induce endogenous oxidative stress. Consistent with our idea, ROS accumulation and oxidative damage to cell structures were observed in *S*. *cerevisiae* wine strains during fermentation in high-sugar-containing medium^11^.

The mitochondrial electron transport chain is thought to be a major source of ROS production, especially when the oxygen reduction reaction is incomplete^12^. The major species of intracellular ROS are superoxide anion (O_2_^•−^), hydrogen peroxide (H_2_O_2_), and hydroxyl radical (^•^OH)^12^. Among these, O_2_^•−^ is the primary and most abundant intracellular ROS^13^. To detoxify O_2_^•−^, SODs are used to disproportionate O_2_^•−^ to H_2_O_2_, which is then further converted into water and O_2_ by catalases^12^. *S*. *cerevisiae* contains two SODs, i.e. cytosolic Cu/Zn-SOD (Sod1p) and mitochondrial Mn-SOD (Sod2p)^14^. In addition to antioxidant enzymes, intracellular ROS accumulation can also be inhibited by supplementation of ROS scavengers, such as N-acetyl-L-cysteine (NAC), ascorbic acid, and glutathione^12,15^.

This study aimed to investigate the effect of fermentation-associated stresses on inducing endogenous oxidative stress. To this end, dynamic changes in intracellular ROS levels during ethanol fermentation were monitored and the role of ROS scavenger supplementation in enhancing performances of VHG ethanol fermentation was evaluated.

## Results

### Cytosolic Cu/Zn-SOD is important for protecting yeast cells against oxidative stress induced by various fermentation-associated stresses

**Figure 1:**
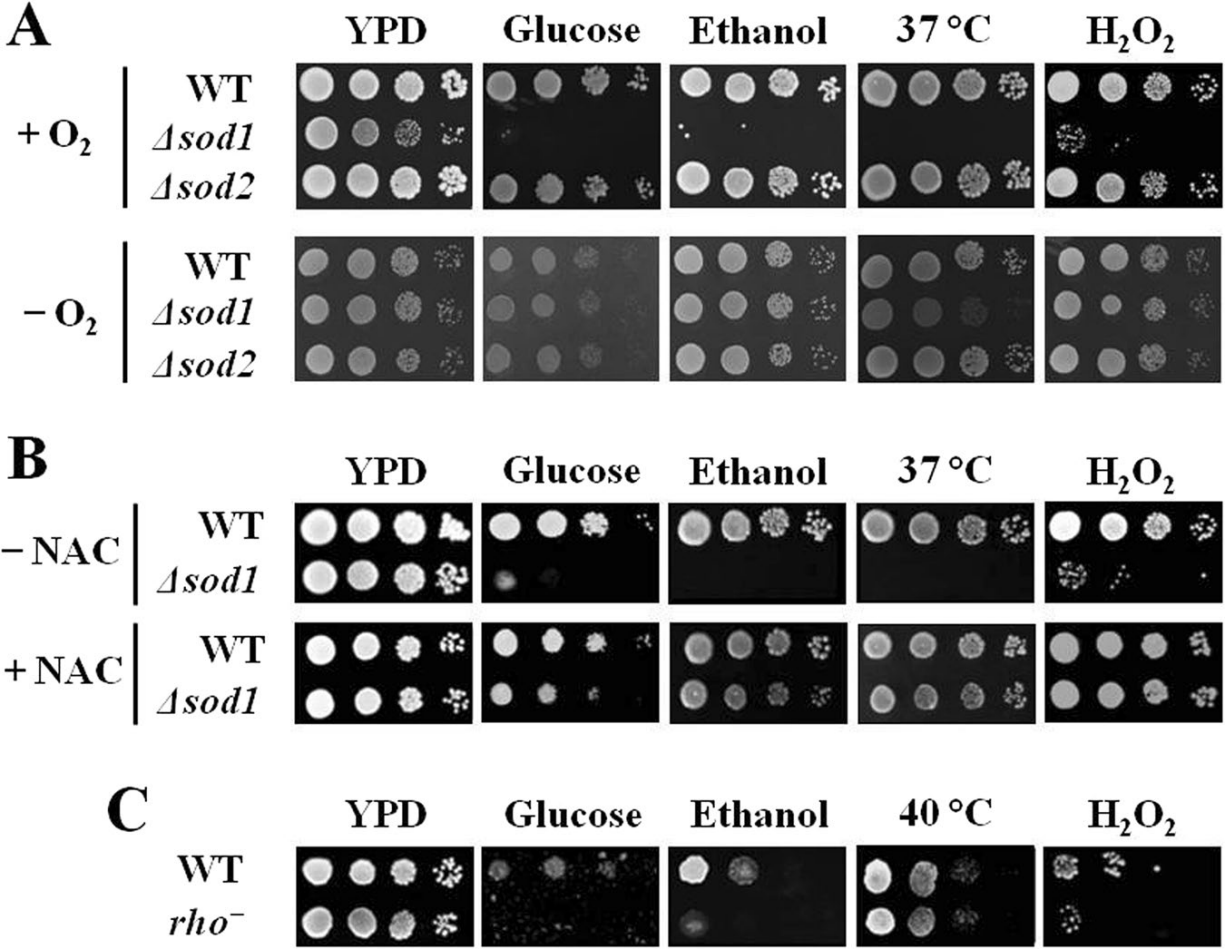
Growth of the wild-type (BY4742), *Δsod1*, and *Δsod2* strains on YPD agar plates containing 30% (w/v) glucose, 10% (v/v) ethanol, or 3 mM H_2_O_2_ at 30 °C or 37 °C (for heat stress) in the presence and absence of (**A**) O_2_ or (**B**) 30 mM N-acetyl-L-cysteine (NAC). (**C**) Growth of the wild-type and *rho*^*−*^ cells on YPD agar plates containing 40% (w/v) glucose, 15% (v/v) ethanol, or 4 mM H_2_O_2_ at 30 °C or 40 °C (for heat stress).

Previously, the *Δsod1* mutant lacking cytosolic Cu/Zn-SOD has been shown to be hypersensitive to various stresses present during ethanol fermentation, including hyperosmotic, ethanol, and heat stresses^9^, suggesting the role of Cu/Zn-SOD in tolerating these stresses. In addition to Cu/Zn-SOD (Sod1p), *S*. *cerevisiae* also contains mitochondrial Mn-SOD encoded by *SOD2* gene^16^. To investigate the role of these two SODs in tolerating various fermentation-associated stresses, we examined aerobic growth of the *Δsod1* and *Δsod2* mutants on YPD agar plates containing 30% (w/v) glucose, 10% (v/v) ethanol, or 3 mM H_2_O_2_ at 30 °C. For heat stress, plates were incubated at 37 °C. Under aerobic condition, the *Δsod1* mutant, but not the *Δsod2* mutant, was hypersensitive to all stresses tested (Fig. 1A), suggesting that only the cytosolic Cu/Zn-SOD is required for tolerating fermentation-associated stresses including osmotic, ethanol, heat and oxidative stresses.

Since cytosolic Cu/Zn-SOD is required for reducing intracellular ROS levels, it is possible that these fermentation-associated stresses may also induce endogenous oxidative stress. To test this hypothesis, we first examined anaerobic growth of the wild-type (BY4742), *Δsod1*, and *Δsod2* strains treated with high glucose, ethanol, H_2_O_2_, and high temperature. We found that the sensitivity of *Δsod1* mutant to all stresses tested was suppressed under anaerobic condition (Fig. 1A). To confirm this hypothesis, we examined the growth of wild-type, *Δsod1*, and *Δsod2* strains on YPD agar plates in the presence of fermentation-associated stresses with or without a supplementation of ROS scavenger, NAC. Consistent with the growth under anaerobic conditions, the growth defect of *Δsod1* mutant was also restored by NAC supplementation (Fig. 1B). These findings therefore suggest that Cu/Zn-SOD is important for protecting yeast cells against cytosolic oxidative damage induced by these fermentation-associated stresses.

### Fermentation-associated stresses induce intracellular ROS production through a mitochondria-independent mechanism

**Figure 2:**
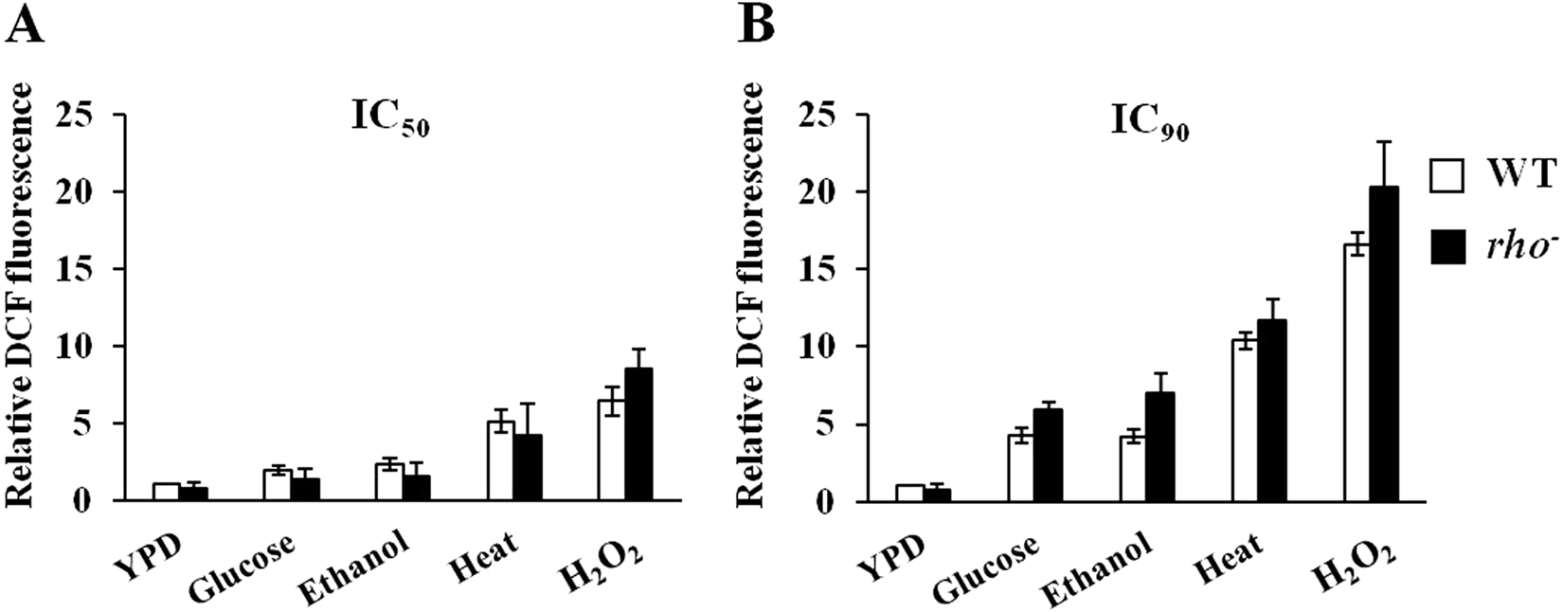
Intracellular ROS levels of the wild-type (BY4742) and *rho*^*−*^ strain after exposures to (**A**) IC_50_ or (**B**) IC_90_ of glucose (20% and 30% (w/v)), ethanol (3% and 7% (v/v)), high temperature (39 °C and 40 °C) or H_2_O_2_ (1 mM and 1.5 mM) for 12 h. Error bars represent ± S.D.

To examine whether these fermentation-associated stresses induce ROS generation, we measured intracellular ROS levels in the wild-type (BY4742) strain after grown in YPD media supplemented with IC_50_ concentrations of glucose (20% (w/v)), ethanol (3% (v/v)) and H_2_O_2_ (1 mM) at 30 °C or in YPD medium at 39 °C, which caused 50% growth inhibition. The intracellular ROS levels were significantly increased after treated with H_2_O_2_ or high temperature (6.4- and 5.1-fold, respectively) and slightly increased after exposures to glucose or ethanol (1.9- and 2.3-fold, respectively) (Fig. 2A). To confirm the effect of these stresses on inducing ROS generation, we determined intracellular ROS levels in the wild-type cells after incubated in YPD media containing IC_90_ concentrations of glucose (30% (w/v)), ethanol (7% (v/v)) and H_2_O_2_ (1.5 mM) at 30 °C, or in YPD medium at 40 °C that inhibited 90% of growth rate. Under these conditions, the intracellular ROS levels were significantly increased after exposures to all stresses tested (Fig. 2B). These results show that osmotic, ethanol, and heat stresses induced ROS production in a concentration-dependent manner.

The mitochondrial electron transport chain is one of the major sources of intracellular ROS^17^. In *S*. *cerevisiae*, several subunits of the electron transport chain are encoded by mitochondrial DNA. When lacking mitochondrial DNA, yeast cells are unable to acquire energy through aerobic respiration but they can survive by using the anaerobic fermentation pathway^18^. If the mitochondrial electron transport chain was the major source of the intracellular ROS produced during exposures to these fermentation-associated stresses, loss of functional mitochondrial respiration is supposed to improve yeast growth under these stress conditions. To determine whether the intracellular ROS induced by these fermentation-associated stresses were mainly generated in the mitochondria, we examined the growth of wild-type strain and BY4742-background *rho*^*−*^ mutant, a respiratory-deficient mutant lacking functional mitochondrial genome, under severe stress conditions (i.e., 40% (w/v) glucose, 15% (v/v) ethanol, 4 mM H_2_O_2_, and 40 °C (for heat stress)). We found that the *rho*^*−*^ mutant did not exhibit an improved growth under all conditions tested, but rather was more sensitive to osmotic, ethanol, and oxidative stresses than the wild-type strain (Fig. 1C). These findings suggest that the mitochondrial respiratory chain may not be the major source of ROS generation induced by these fermentation-associated stresses. To further assess whether the mitochondrial metabolism is involved in the induction of intracellular ROS generation upon exposures to these fermentation-associated stresses, we determined the ROS levels in the *rho*^*−*^ mutant treated with IC_50_ and IC_90_ values of the wild-type strain for each stress (i.e., glucose (20% and 30% (w/v)), ethanol (3% and 7% (v/v)), high temperature (39 °C and 40 °C), and H_2_O_2_ (1 mM and 1.5 mM)). Under all stress conditions tested, the ROS levels in the *rho*^*−*^ mutant were similar to those of the wild-type strain (Fig. 2A,B). These results support our idea that the fermentation-associated stresses (i.e., osmotic, ethanol, heat, and oxidative stresses) promote ROS generation through a mitochondria-independent mechanism.

### Cu/Zn-SOD is required for reducing cytosolic ROS levels induced by fermentation-associated stresses

**Figure 3:**
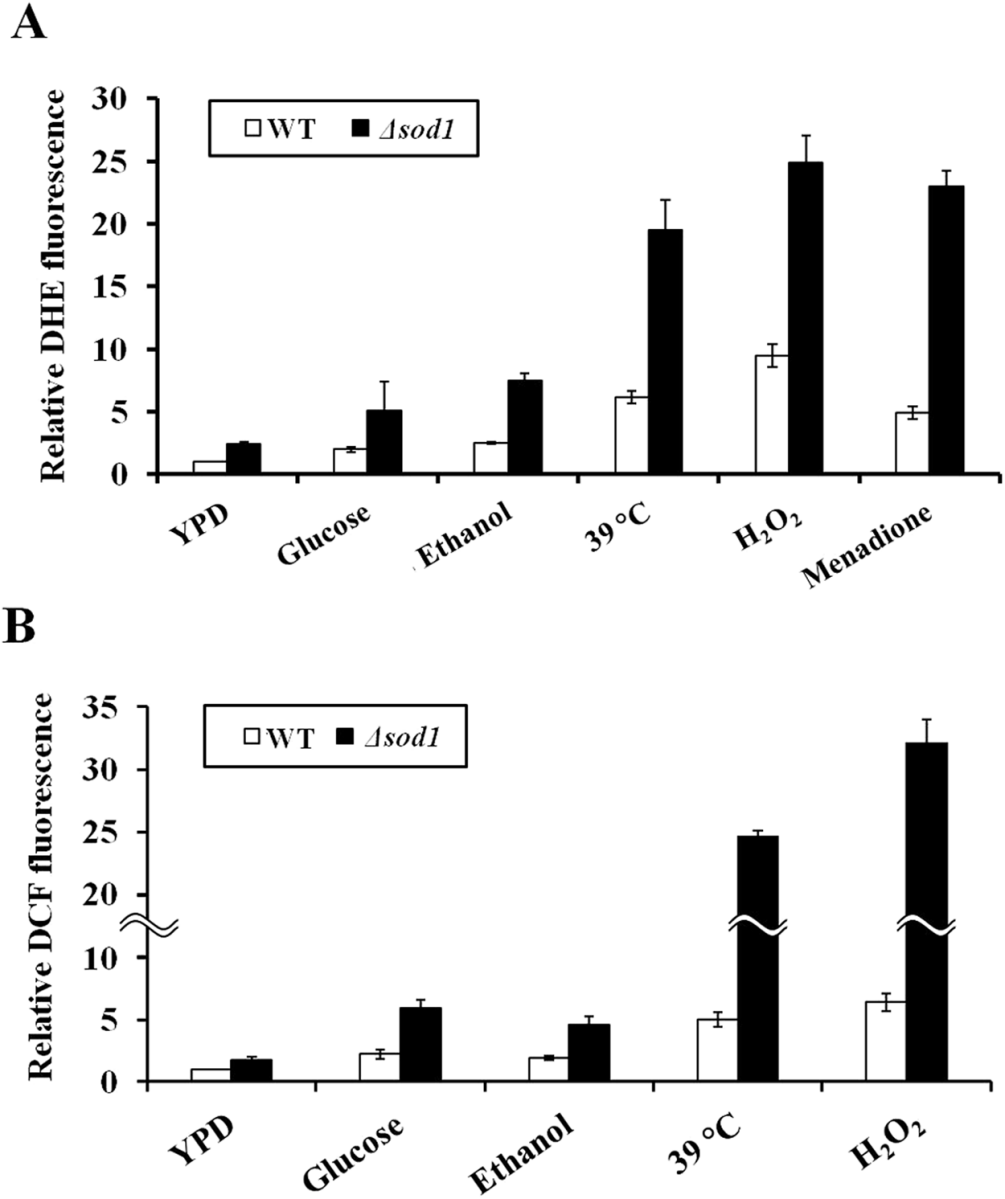
Intracellular (**A**) O_2_^•−^ and (**B**) ROS levels of the wild-type (BY4742) and *Δsod1* strains after exposures to 20% (w/v) glucose, 3% (v/v) ethanol, or 1 mM H_2_O_2_ at 30 °C or 39 °C (for heat stress) for 12 h. Error bars represent ± S.D.

Since Cu/Zn-SOD is an antioxidant enzyme that catalyzes the disproportionation of cytosolic O_2_^•−^ to H_2_O_2_, it is possible that hypersensitivity of the *Δsod1* mutant to these fermentation-associated stresses may be due to a hyperaccumulation of cytosolic O_2_^•−^ and other radicals. To test this hypothesis, we measured the intracellular O_2_^•–^ and ROS levels in the wild-type and *Δsod1* strains exposed to high glucose, ethanol, H_2_O_2_, menadione (an O_2_^•−^ generator), or high temperature. In the wild-type (BY4742) strain, the levels of intracellular O_2_^•−^ were significantly increased in response to all stresses, especially after exposed to high temperature and H_2_O_2_ (Fig. 3A). Under all stress conditions tested, the intracellular O_2_^•−^ levels of the *Δsod1* mutant were significantly higher than those of the wild-type strain (Fig. 3A). Consistent with the O_2_^•−^ levels, the total intracellular ROS levels in the *Δsod1* mutant were also significantly higher than those of the wild-type strain after exposures to these fermentation-associated stresses (Fig. 3B). These results suggest the role of Cu/Zn-SOD in reducing cytosolic O_2_^•−^ levels induced by fermentation-associated stresses, which in turn reduces intracellular ROS levels.

### ROS scavenger alleviates endogenous oxidative stress induced during VHG fermentation

**Figure 4:**
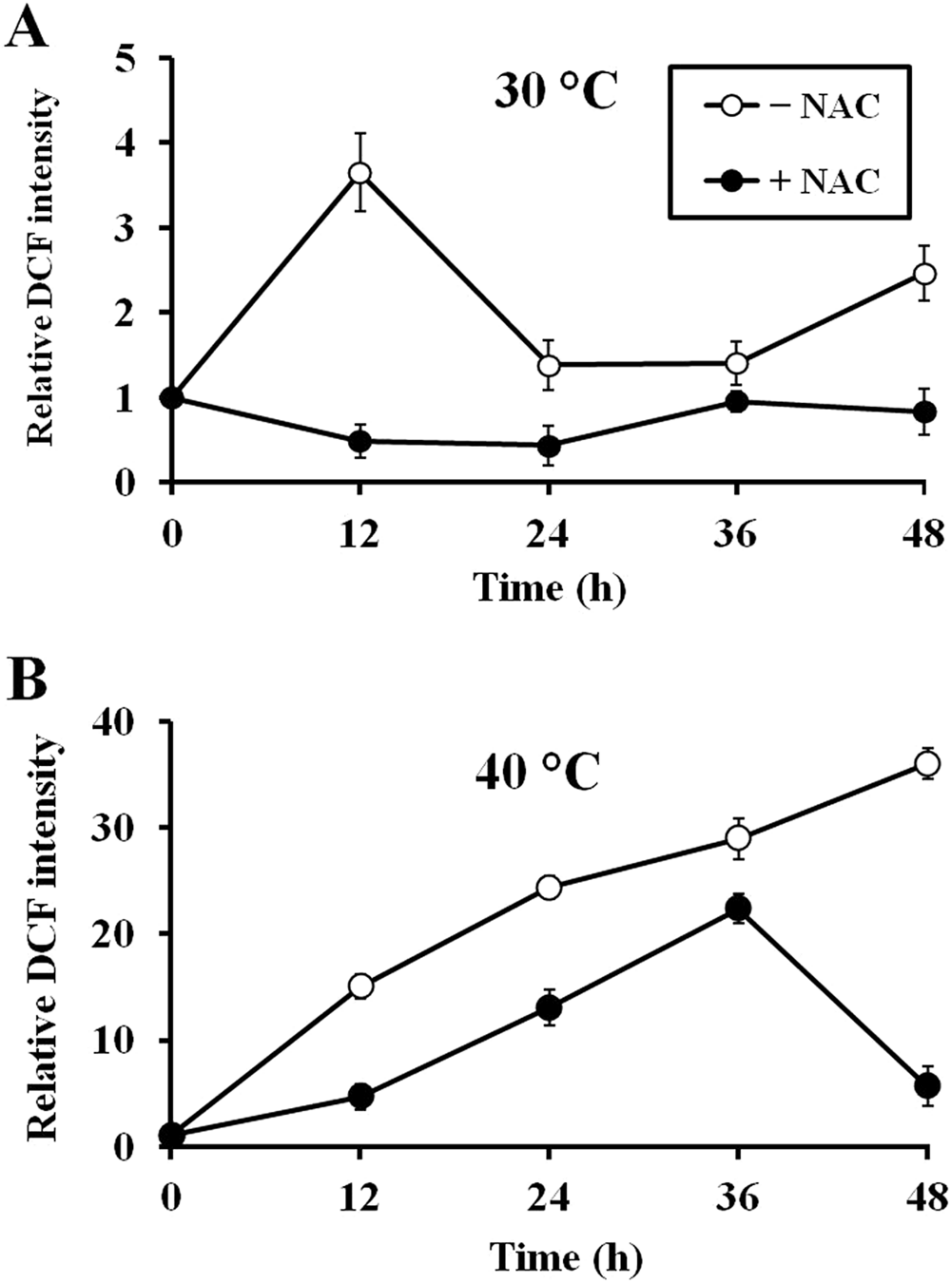
Intracellular ROS levels of the wild-type (BY4742) strain during fermentation in YPD30 media in the presence and absence of 30 mM NAC for 48 h at (**A**) 30 °C or (**B**) 40 °C. Error bars represent ± S.D.

Since ROS accumulation and oxidative damage to yeast cell structures have been observed during fermentation^11^, we monitored changes of intracellular ROS levels in the wild-type (BY4742) strain incubated in YPD30 media (YPD media containing 30% (w/v) glucose) at 30 and 40 °C in the presence and absence of ROS scavenger, NAC. In the absence of NAC, the intracellular ROS levels in the wild-type strain incubated at 30 °C were remarkably increased after 12 h-incubation and significantly decreased after incubation for more than 24 h (Fig. 4A), while incubation at high temperature (40 °C) resulted in a dramatic and continuous increase of intracellular ROS levels (Fig. 4B). When NAC was applied, the intracellular ROS levels in the wild-type strain incubated at 30 °C were maintained at basal level throughout the incubation (Fig. 4A). Likewise, at 40 °C, the ROS levels of the wild-type strain incubated in the presence of NAC were significantly lower than those without NAC supplementation (Fig. 4B). These results suggest the role of ROS scavengers such as NAC in protecting yeast cell against oxidative stress induced during VHG fermentation at both normal and high temperatures, thereby contributing to the maintenance of yeast metabolism.

### ROS scavenger plays an important role in maintaining Cu/Zn-SOD activity during VHG fermentation

**Figure 5:**
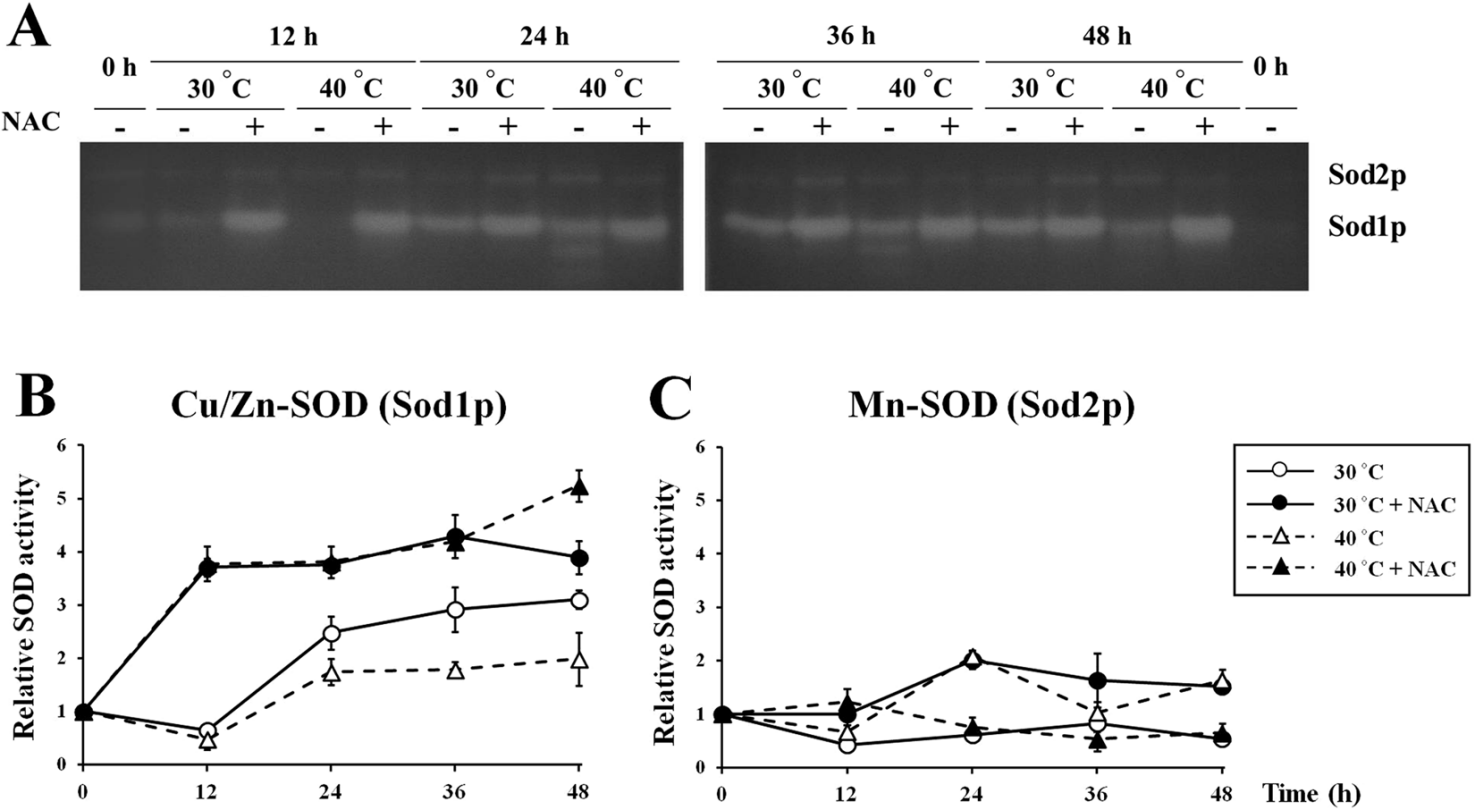
Cu/Zn-SOD and Mn-SOD activities of the wild-type (BY4742) strain during fermentation in YPD30 media in the presence and absence of 30 mM NAC for 48 h at 30 °C or 40 °C. (**A**) The activities of Cu/Zn-SOD and Mn-SOD were analyzed by using native gel electrophoresis and nitroblue tetrazolium staining. Quantitation of the Cu/Zn-SOD and Mn-SOD activity bands of the wild-type (BY4742) strain incubated at (**B**) 30 °C or (**C**) 40 °C was performed with ImageJ software. Error bars represent ± S.D.

Since our data suggest that Cu/Zn-SOD is required for protecting yeast cells against cytosolic ROS induced by fermentation-associated stresses, we next investigated the role of Cu/Zn-SOD during VHG fermentation by monitoring Cu/Zn-SOD (Sod1p) and Mn-SOD (Sod2p) activities in the wild-type (BY4742) cells incubated in YPD30 media at 30 and 40 °C in the presence and absence of NAC. At both temperatures, Cu/Zn-SOD activity in the wild-type cells treated with NAC was significantly increased within 12-h incubation and maintained at high levels throughout the fermentation (Fig. 5A,B). On the other hand, Cu/Zn-SOD activity of those incubated in the absence of NAC was inhibited during the first 12 h of incubation (Fig. 5A,B). Although, after 12-h incubation, an increase of Cu/Zn-SOD activity was observed in the wild-type cells incubated without NAC, their levels of Cu/Zn-SOD activity were lower than those treated with NAC (Fig. 5A,B). These findings therefore suggest the critical role of ROS scavenger in maintaining Cu/Zn-SOD activity during VHG fermentation. In the case of mitochondrial Mn-SOD activity, we found that, irrespective of NAC supplementation, its activity was not significantly induced during VHG fermentation at both temperatures (Fig. 5A,C). These results support our concept that only Cu/Zn-SOD, but not Mn-SOD, plays an important role in protecting yeast cells against cytosolic ROS induced by fermentation-associated stresses.

### ROS scavenger enhances VHG fermentation performance

**Figure 6:**
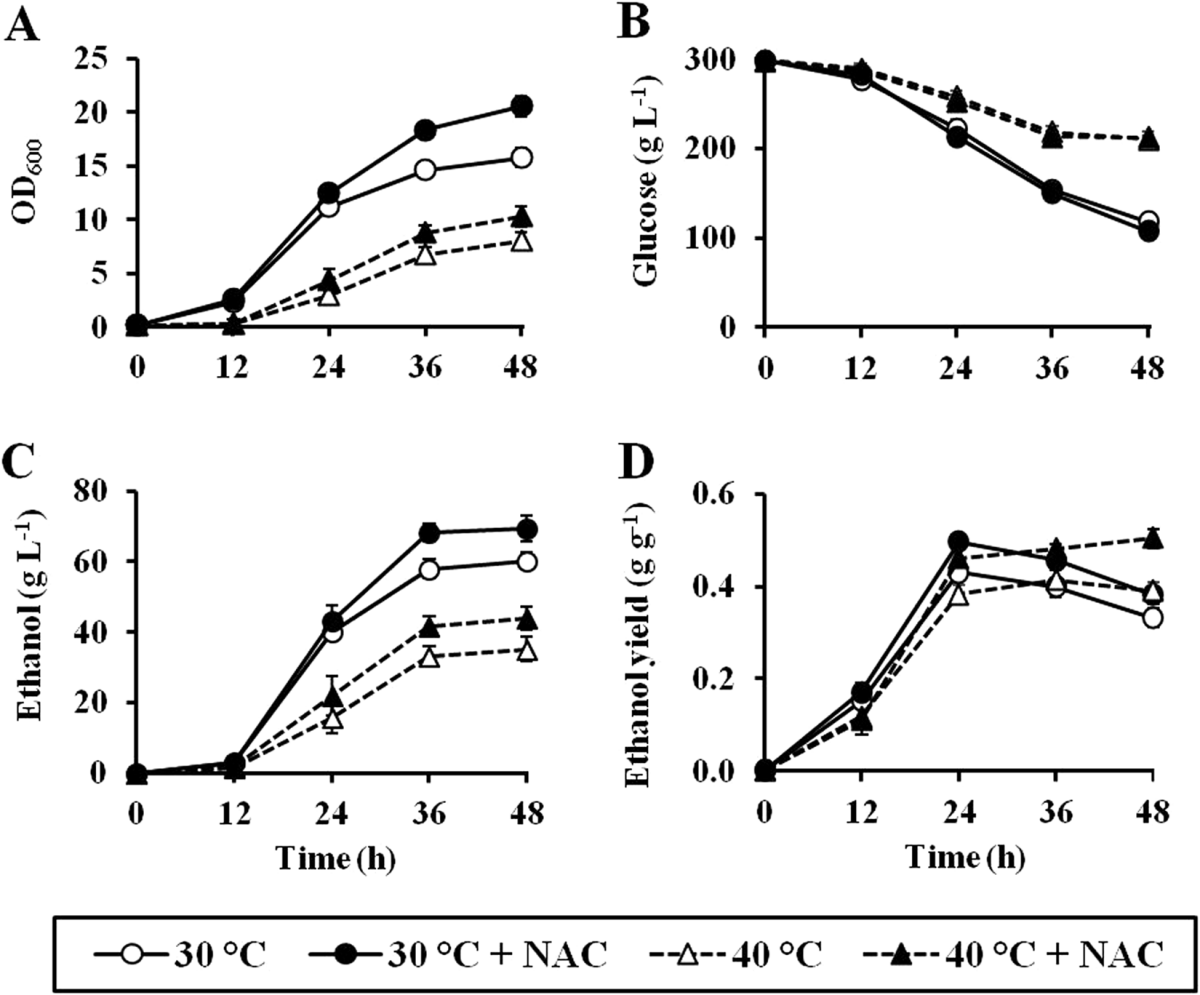
Ethanol fermentation performances of the wild-type (BY4742) in YPD30 media in the presence and absence of 30 mM NAC at 30 °C or 40 °C for 48 h. (**A**) Growth, (**B**) glucose consumption, (**C**) ethanol production, and (**D**) ethanol production yield were determined. Error bars represent ± S.D.

Since our results suggest the effect of ROS scavengers such as NAC on inhibiting endogenous oxidative stress induced during VHG fermentation, we then investigated the role of NAC in promoting VHG fermentation performance of the wild-type (BY4742) strain incubated in YPD30 media at 30 and 40 °C up to 48 h. As expected, due to the effect of heat stress, the growth rates of wild-type strain incubated at 40 °C were lower than those incubated at 30 °C. At both incubation temperatures, the addition of NAC increased the growth rates of the wild-type strain (Fig. 6A), supporting the idea that NAC is important for protecting yeast cells against oxidative damage occurring during VHG fermentation. At both 30 and 40 °C, although NAC supplementation had no effect on the glucose consumption rates of the wild-type strain (Fig. 6B), the wild-type cells incubated with NAC produced higher ethanol levels (69.3 and 44.0 g L^−1^ of ethanol, respectively) than those without NAC (60.3 and 35.2 g L^−1^ of ethanol, respectively) (Fig. 6C). Moreover, the NAC supplementation enhanced the ethanol yields of the wild-type strain up to 0.50 g ethanol/g glucose consumed, after incubation for 24 h at 30 °C or for 48 h at 40 °C (Fig. 6D). Our results therefore suggest a potential role of ROS scavenger for improving ethanol production in VHG fermentation.

**Figure 7:**
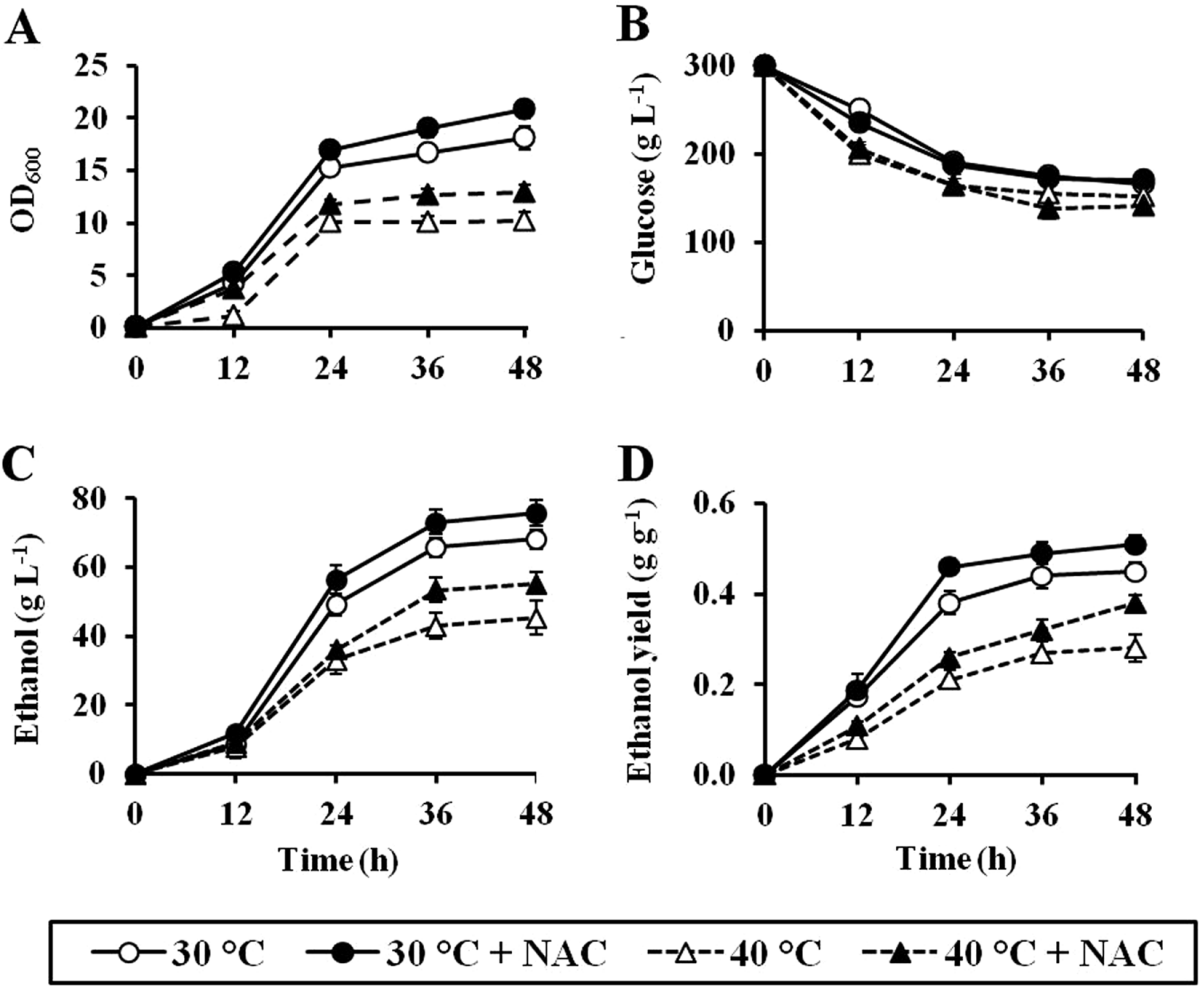
Ethanol fermentation performances of the industrial strain (TISTR5606) in YPD30 media in the presence and absence of 30 mM NAC at 30 °C or 40 °C for 48 h. (**A**) Growth, (**B**) glucose consumption, (**C**) ethanol production, and (**D**) ethanol production yield were determined. Error bars represent ± S.D.

To further investigate the potential of ROS scavengers such as NAC for application in industrial VHG ethanol fermentation, we next monitored the ethanol fermentation performance of the industrial ethanol-producing strain (TISTR5606) incubated in YPD30 media in the presence and absence of NAC at 30 and 40 °C up to 48 h. Similar to the case of the laboratory stain BY4742, the addition of NAC also enhanced the growth rates, the ethanol productivities, and the ethanol yields of the industrial TISTR5606 strain incubated at both temperatures (Fig. 7A–D). After 48-h incubation at 30 and 40 °C without NAC supplementation, the TISTR5606 strain produced 66.1 and 45.4 g L^−1^ of ethanol to give ethanol yields of 0.45 and 0.28 g ethanol/g glucose consumed, respectively (Fig. 7C,D). By the supplementation with NAC, the ethanol productivities of TISTR5606 strain after fermentation for 48 h at 30 and 40 °C were increased to 73.6 and 55.3 g L^−1^, respectively, which in turn leads to an improvement in its ethanol yields to 0.50 and 0.38 g ethanol/g glucose consumed, respectively (Fig. 7C,D). These results further confirm that ROS scavenger has high potential for use to improve ethanol fermentation performance of industrial-scale VHG fermentation at both normal and high temperatures.

## Discussion

In this study, we show the effects of fermentation-associated stresses, i.e. hyperosmolarity, high ethanol concentration, and high temperature, on promoting ROS generation in the cytosol. In addition, we also found that intracellular ROS were highly accumulated during VHG fermentation at both normal and high temperatures. Consistent with our results, it has been reported that increased ROS accumulations were observed after ethanol treatment and during fermentation of high-sugar-containing medium^10,11,19^. Endogenous oxidative stress can be directly induced by pro-oxidant activities of some chemical compounds such as 2,4-dichlorophenoxy acetic acid (2,4-D) or be indirectly caused by the protein denaturing effect of chemical agents such as alachlor^20^. For heat stress, it is well-known to severely disrupt protein structure, leading to an induction of several heat stress responses including chaperone-mediated protein folding in order to prevent protein denaturation and aggregation^21^. Likewise, ethanol has been reported to induce protein damage and trigger the unfolded protein response^5,22^. In addition, previous results of microarray analysis showed that genes involved in antioxidant defense and heat shock response were up-regulated upon exposures to ethanol and high temperature^23,24^. Taken together, it is likely that the protein-denaturing effects of ethanol and heat stresses may be the cause of the increased ROS production during exposures to these stresses. Since some antioxidant proteins such as Gpx2p and Trx1p have been reported to be down-regulated in response to ethanol and heat stresses, respectively^25^, a decrease of intracellular antioxidants may also contribute to an increase in ROS accumulation under stress conditions. Previously, it has been shown that the synthesis of trehalose, a disaccharide involved in protecting intracellular proteins against damage and denaturation through its function as a protein stabilizer^26^, was inhibited under high glucose condition^27,28^. It is therefore possible that, due to a loss of trehalose-mediated protein stabilization, the high glucose stress might also cause protein denaturation, which in turn elevates intracellular ROS levels.

Although the mitochondrial electron transport chain is thought to be a major source of intracellular ROS, our results obtained from the *rho*^*−*^ mutant revealed that osmotic, ethanol, and heat stresses promoted generation of intracellular ROS through mitochondria-independent mechanisms. In agreement, we found that cytosolic Cu/Zn-SOD, but not mitochondrial Mn-SOD, was required for protecting yeast cells against oxidative damage induced by these fermentation-associated stresses through its role in reducing intracellular O_2_^•−^. Nevertheless, the activity of Cu/Zn-SOD seems to be partially inhibited during early stages of ethanol fermentation, possibly by the effect of increased ROS generation induced by fermentation-associated stresses. Based on these results, it is likely that various fermentation-associated stresses, including osmotic, ethanol, and heat stresses, may primarily induce protein denaturation and inhibit activities of antioxidant enzymes such as Cu/Zn-SOD, which in turn promotes ROS generation in the cytosol through the mitochondrial-independent mechanism. Interestingly, we found that although the treatment with NAC as a ROS scavenger resulted in reduced intracellular ROS levels, high Cu/Zn-SOD activity was still observed in the NAC-treated cells. This may be due to the fact that, during fermentation, yeast cells are continuously exposed to several fermentation-associated stresses that enhance ROS production. For this reason, it seems that activities of some intracellular antioxidants including Cu/Zn-SOD are required to be maintained at constant levels throughout fermentation. Previously, it has been reported that a NADPH oxidase-like protein (Yno1p) localized to the ER membrane is the extra-mitochondrial source of ROS in *S*. *cerevisiae*, due to its effect on catalyzing the production of O_2_^•−^ from oxygen and NADPH^29^. It is therefore possible that some intracellular ROS induced by these fermentation-associated stresses might be also partially mediated through Yno1p.

Interestingly, we found that the *rho*^*−*^ mutant with extensive deletions of mitochondrial DNA was more sensitive to osmotic, ethanol, and oxidative stresses than the wild-type strain. These results suggest that the increased sensitivity of *rho*^*−*^ mutant to these stresses might be caused by some other defects in this mutant. Previously, it has been reported that the *rho* mutation affects peripheral, biologically active glycoproteins (such as cell wall or extracellular enzymes) and structural glycoproteins (such as peripheral phosphopeptidomannans (PPMs)), leading to severe defects in cell wall biosynthesis and cell-cell recognition process^30^. In addition, a loss of mitochondrial DNA has been shown to result in unusual mitochondrial morphology^31^ and defective intergenomic signaling, which functions in coordinating mitochondrial and nuclear gene expression^32^. Taken together, it is possible that the lack of complete mitochondrial genome may lead to multiple cellular defects, which in turn increase sensitivity to several stresses, including osmotic, ethanol, and oxidative stresses.

Finally, we demonstrate that the reduction of endogenous oxidative stress by the supplementation of ROS scavengers such as NAC improved ethanol fermentation performances of both laboratory (BY4742) and industrial (TISTR5606) yeast strains in VHG fermentation. Consistent with our results, it has been reported that improvement of catalase activity, one of important antioxidant enzymes, by supplementation of biotin resulted in an increase of ethanol productivity^33^. Based on our findings, it appears that oxidative stress is another major stress occurring during VHG fermentation, and the supplementation of antioxidants as ROS scavengers have high potential for improving an efficiency of ethanol production on industrial-scale VHG fermentation at both normal and high temperatures. Nevertheless, NAC seems to be inappropriate for use in industrial-scale VHG ethanol fermentation due to its cost-ineffectiveness. From this point of view, cheaper antioxidants such as ascorbic acid or plant-based natural antioxidants appear to be more suitable. However, the potential of these candidate antioxidants for application in VHG ethanol fermentation is needed to be evaluated. In addition to antioxidant supplementation, optimal aeration has been reported to be essential to achieve high ethanol yield under VHG fermentation conditions, due to its role in maintaining a proper oxygen supply and a stable redox environment^34^. However, aeration will also increase oxygen solubility in fermentation media, thereby potentially enhancing ROS production. Therefore, to achieve highly efficient ethanol production, VHG fermentation with a controlled aeration and antioxidant supplementation is required. Furthermore, it should be noted that a considerable amount of glucose was remained unutilized in the media at the end of ethanol fermentation by the laboratory (BY4742) or industrial (TISTR5606) yeast strains. To obtain maximum fermentation efficiency, an optimization of glucose concentration and/or other factors is needed to be further studied.

In conclusion, we found that several stresses occurring during ethanol fermentation, i.e. glucose-induced hyperosmolarity, high ethanol concentration, and high temperature, induced endogenous oxidative stress in *S*. *cerevisiae* cells by enhancing the production of intracellular ROS such as O_2_^•−^ through mitochondria-independent pathways. To cope with oxidative stress induced by these fermentation-associated stresses, cytosolic Cu/Zn-SOD, but not mitochondrial Mn-SOD, is essential for reducing intracellular O_2_^•−^. Furthermore, supplementation of ROS scavengers such as NAC into fermentation media promoted not only reduction of intracellular ROS levels but also efficient ethanol production, suggesting a high potential of ROS scavengers for application in industrial ethanol fermentation.

## Materials and Methods

### Yeast strains and growth conditions

The *S*. *cerevisiae* strains used in this study were the wild-type BY4742 (*MATα his3Δ1 leu2Δ0 lys2Δ0 uraΔ0*) and its isogenic mutants, i.e. *Δsod1*::*kanMX* and *Δsod2*::*kanMX* (EUROSCARF), [*rho*^*−*^] LJ104^35^, and TISTR5606 (Thailand Institute of Scientific and Technological Research). YPD medium (1% yeast extract, 2% peptone, 2% dextrose) and YPD30 medium (YPD medium containing 30% glucose) were prepared as described previously^36,37^.

### Spot susceptibility assay

Log-phase cells were harvested, resuspended in sterile water to an OD_600_ of 1.0, and then serially diluted ten-fold. An aliquot (3 µL) of each dilution sample was spotted onto YPD agar plates and YPD agar plates containing glucose, ethanol, or H_2_O_2_ with and without the addition of 30 mM N-acetyl-L-cysteine (NAC). The plates were incubated at 30 °C or 37–40 °C (for growth determination at high temperature) for three days under aerobic and anaerobic conditions as described previously^38^. Briefly, for growth under anaerobic condition, the plates were incubated in an AnaeroPack Rectangular Jar (Mitsubishi Gas Chemical Co., Tokyo, Japan) containing an AnaeroPack working as an oxygen absorber-CO_2_ generator (Mitsubishi Gas Chemical Co., Tokyo, Japan).

### Measurement of intracellular ROS

The oxidant-sensitive fluorescent probe 2′,7′-dichlorofluorescein diacetate (DCFH-DA; Sigma) was used to determine the intracellular ROS levels as described previously^38^. Log-phase cells were treated with 10 mM DCFH-DA in culture media for 1 h. Cells were then harvested, washed, resuspended in PBS buffer, and disrupted with glass beads. The supernatants were collected by centrifugation at 13,000 × *g* for 10 min at 4 °C. The fluorescence intensities were measured by a microplate reader (Tecan Infinite, M200 Pro) at an excitation wavelength of 490 nm and an emission wavelength of 524 nm. The fluorescence intensity values were normalized to protein levels in the supernatants.

### Measurement of intracellular O_2_^•−^

Intracellular O_2_^•−^ levels were determined by using dihydroethidium (DHE; Sigma) as described previously^20^. Briefly, 5 × 10^6^ log-phase cells were incubated with 50 µM DHE in YPD media for 15 min at 30 °C in the dark. Cells were then collected by centrifugation at 3,000 × *g* for 5 min at 4 °C, and washed with PBS buffer. The fluorescence intensities were measured by using a microplate reader (Tecan Infinite, M200 Pro) at an excitation wavelength of 535 nm and an emission wavelength of 635 nm. The fluorescence intensity values for each sample were normalized by the cell density (OD_600_).

### Analysis of SOD activity

Log-phase wild-type (BY4742) cells were inoculated into 250 mL Erlenmeyer flask containing 50 mL of YPD30 medium at the initial cell density (OD_600_) of 0.1. The flasks were sealed with parafilm to mimic semi-fermentative conditions and incubated with shaking at 30 or 40 °C for 48 h. At the indicated times, the samples were collected and centrifuged at 13,000 × *g* for 3 min. Whole cell lysates were prepared by glass bead homogenization^39^ in 0.5 ml lysis buffer (10 mM NaHPO_4_ (pH 7.8), 5 mM EDTA, 5 mM EGTA, 0.1% Triton-X, 50 mM NaCl, 300 mM sorbitol, 0.5 mM phenylmethylsulfonyl fluoride (PMSF) and Protease Inhibitor cocktail (Sigma)). The lysates were analyzed for SOD activity by native gel electrophoresis and nitroblue tetrazolium staining as described previously^40^. Quantitation was performed with ImageJ software (Rasband, W. S., NIH, USA)

### VHG ethanol fermentation

Log-phase cells were inoculated into 250 mL Erlenmeyer flask containing 50 mL of YPD30 medium at the initial cell density (OD_600_) of 0.1. The flasks were sealed with parafilm to allow fermentation to be carried out under semi-anaerobic conditions. For normal and high temperature fermentations, flasks were incubated with shaking at 30 or 40 °C, respectively, for 48 h. The fermentation samples were collected by centrifugation at 13,000 × *g* for 3 min. Ethanol and glucose concentrations were determined by using the ethanol assay F-kit (Roche Diagnostics, Basel, Switzerland) and the glucose assay F-kit (Roche Diagnostics, Basel, Switzerland), respectively.

### Data analysis

All experiments were independently performed at least three times and expressed as means with standard deviations. Analysis of variance was performed by one-way analysis of variance (ANOVA) using least significant difference method (LSD) on the SPSS statistical package (version 18.0 for Windows, SPSS Inc., Chicago, IL, USA). The statistical significance was set at *p* < 0.05.

## Electronic supplementary material

Supplementary Information
