# Supplementary material for: Enhancement of ethanol production in very high gravity fermentation by reducing fermentation-induced oxidative stress in Saccharomyces cerevisiae
Source: Sci Rep. 2018 Aug 30;8:13069. doi: 10.1038/s41598-018-31558-4 (PMC6117276; doi:10.1038/s41598-018-31558-4)
Supplement: Supplementary file 1 — Supplementary Information [file 41598_2018_31558_MOESM1_ESM.pdf]

Enhancement of ethanol production in very high gravity fermentation by reducing fermentation-induced oxidative stress in *Saccharomyces cerevisiae*

Thanawat Burphan[1,2], Supinda Tatip[1,2], Tossapol Limcharoensuk[1,2], Kitsada Kangboonruang[1], Chuenchit Boonchird[3], Choowong Auesukaree[1,2,3]*

[1]Department of Biology, Faculty of Science, Mahidol University, Bangkok 10400, Thailand
[2]Center of Excellence on Environmental Health and Toxicology, CHE, Ministry of Education, Bangkok 10400, Thailand
[3]Department of Biotechnology, Faculty of Science, Mahidol University, Bangkok 10400, Thailand

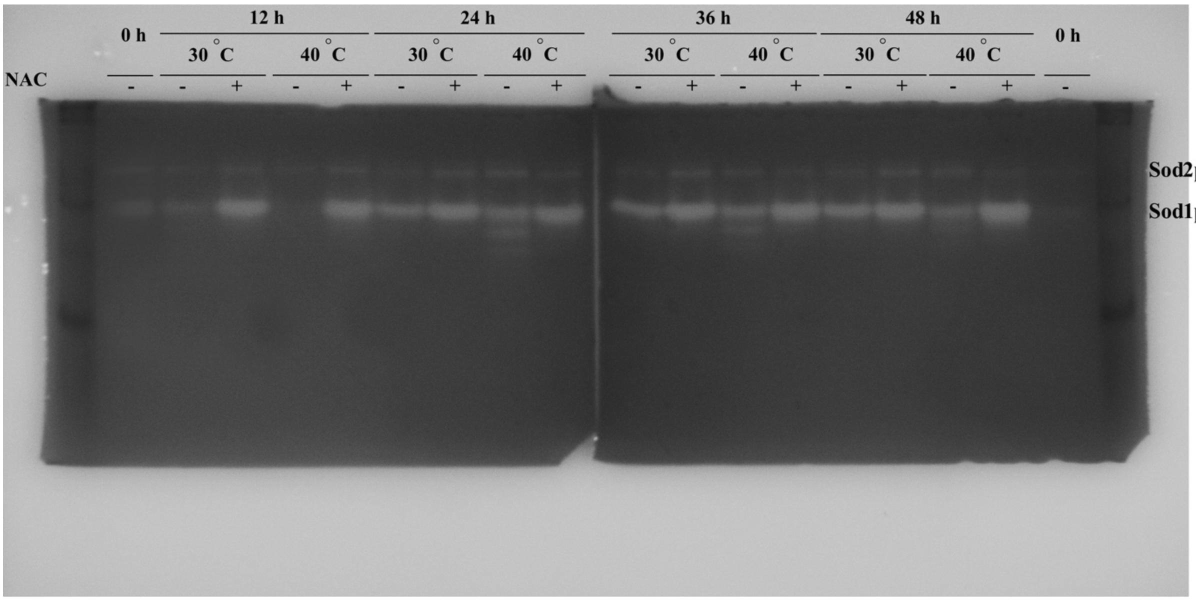

Fig. 5A
